# Computational Methods in Immunology and Vaccinology: Design and Development of Antibodies and Immunogens

**DOI:** 10.1021/acs.jctc.3c00513

**Published:** 2023-08-01

**Authors:** Federica Guarra, Giorgio Colombo

**Affiliations:** Department of Chemistry, University of Pavia, Via Taramelli 12, 27100 Pavia, Italy

## Abstract

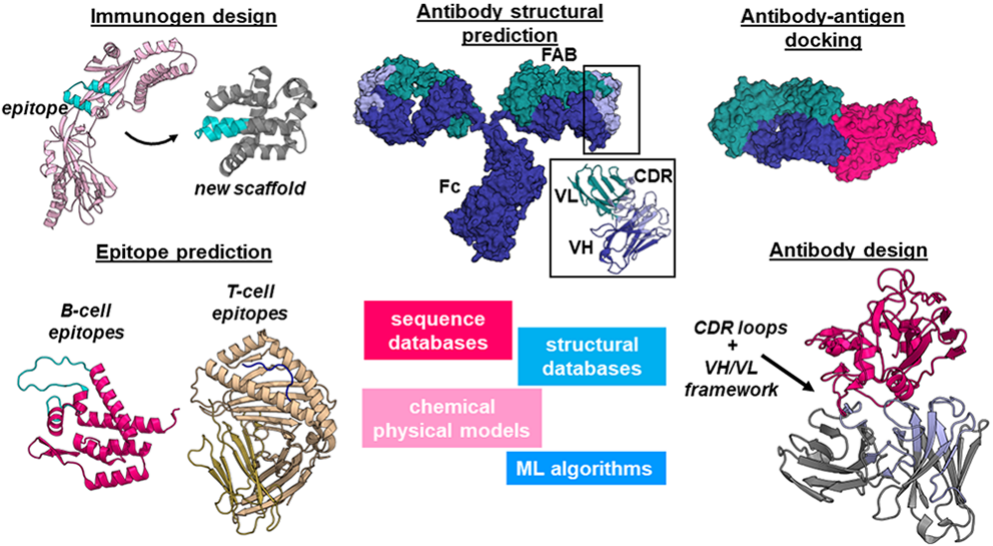

The design of new biomolecules able to harness immune mechanisms for the treatment of diseases is a prime challenge for computational and simulative approaches. For instance, in recent years, antibodies have emerged as an important class of therapeutics against a spectrum of pathologies. In cancer, immune-inspired approaches are witnessing a surge thanks to a better understanding of tumor-associated antigens and the mechanisms of their engagement or evasion from the human immune system. Here, we provide a summary of the main state-of-the-art computational approaches that are used to design antibodies and antigens, and in parallel, we review key methodologies for epitope identification for both B- and T-cell mediated responses. A special focus is devoted to the description of structure- and physics-based models, privileged over purely sequence-based approaches. We discuss the implications of novel methods in engineering biomolecules with tailored immunological properties for possible therapeutic uses. Finally, we highlight the extraordinary challenges and opportunities presented by the possible integration of structure- and physics-based methods with emerging Artificial Intelligence technologies for the prediction and design of novel antigens, epitopes, and antibodies.

## 1 Introduction

The rational development of new strategies to control cellular networks is a key challenge and a trove of opportunities for the scientific community.

The ways and mechanisms through which proteins interact are what shape such networks and determine their functions. The levels of involved proteins, their interaction strengths, as well as alterations in interaction partners build network connectivity at a proteome-wide scale ultimately defining cell phenotypes.^1,2^

Such phenomena are epitomized by mechanisms by which protein–protein recognition, interactions, and (re)organizations shape the functional networks that control immune responses.^3−6^

In this general framework, molecular designers have the opportunity to address general and specific issues that will have important returns in the development of new diagnostics and therapeutics with potential in personalized medicine.

Vaccination is arguably the most successful medical discovery that has benefited human health globally. Worldwide vaccination campaigns have contained the recent Covid-19 pandemic and eradicated life-threatening diseases.^7−9^ The development of new immunotherapies is now realizing its potential in combating drug resistance.^10^ Moreover, exciting results are reported in the treatment of cancer, where immunooncology is becoming an increasingly important tool for patients. In this context, the discovery of the mechanisms of negative immune regulation and approval of immune checkpoint inhibitors had a primary role in revealing the potentialities of the immune system cells in counteracting tumor development,^11,12^ and the prognostic value of the tumor-associated immune landscape is now becoming more and more apparent.^13^ More recently, chimeric antigen receptor-based therapies (CAR-T) were approved for the treatment of some hematologic cancers (acute lymphoblastic leukemia, multiple myeloma, and different type of lymphomas)^14,15^ while showing clinically significant antitumor activities in other malignancies.^16,17^ Interestingly, in these therapies, the unpaired targeting specificity of monoclonal antibodies (mAbs) is combined with the cytotoxicity and long-term persistence provided of T-cells.

Following the genome era advent, conventional immunology has been overtaken by a more rapid and effective *in silico*-led approach to protein-antigen (Ag) selection, termed Reverse Vaccinology (RV), and subsequently, pan-genomic RV,^18,19^ which resulted in the first vaccine, Bexsero (approved by the European Medicines Agency in 2013), against meningococcal serotype B infections.^20^ The most recent development of RV, termed Structural Vaccinology (SV), exploits atomic-level three-dimensional information to engineer Ags with improved immunological and/or biochemical properties.^21^

In a time where medical challenges are numerous, the value of being able to design protein antigens and antibodies with rational methods is clearly manifold. Besides providing new approaches to diagnostics and therapeutics development, this will generate new concepts and methods of general applicability in the realm of protein-interaction studies in health and disease, favoring the development of novel precision tools for chemical and cell biology.

In a different context, the possibility to design protein binders that may act as therapeutic tools, engaging specific molecular targets and hijacking pathologic protein pathway, has the potential to provide novel approaches to block the detrimental activities of proteins that still remain largely undruggable by classical interventions.^22,23^ One particularly interesting field of application entails the manipulation of the activation of cell-surface receptors in response to extracellular signals. Proteins can, in fact, be designed to engage the extracellular domains of these receptors, in some cases driving their clustering on the cell surface, and ultimately generating different downstream cell cascades different from the ones observable in the absence of the binder protein.^24,25^

Our ability to design molecules that target proteins in specific networks thus has important implications for basic science and applied research in chemical biology, biochemistry, and molecular medicine. From the fundamental point of view, designed biomolecules can help advance our understanding of key interactions for diseases (which entail both host–pathogen interactions and derailed networks in nontransmissible pathologies) at the atomic level. Indeed, selective perturbation of well-defined complexes can aptly translate into an impact on the functional activities of the involved biomolecules, shedding light on their roles in biochemical pathways. From an applicative point of view, this knowledge can be translated into new rules for the efficient discovery of new candidates, such as antigens for vaccine development, proteins as biological therapeutics, and new molecular probes for diagnostic development.

To make progress along this avenue, it is first necessary to understand what makes a protein substructure a potential (immune) interaction surface and then translate this information into the design of new systems that mimic desired interactions.

This is an exciting time for this field of research: fundamental contributions have been presented over the past few years in the structure-based design of new antigens, antibodies, and binders in general. The advent of Artificial Intelligence (AI) now enables protein structure prediction and analysis on an unprecedented scale and with accuracy. It is clear that the combination of AI with biophysics- and biochemistry-based methods will shape the next few years, speeding up the discovery of novel biologics with a wide range of applications.

Here, we will review some of the main advancements in these fields with a specific focus on methods involving the development and application of physical chemistry and computational chemistry approaches. A more general overview of methods relying on bioinformatic (and AI-powered) tools is provided, and, where needed, the reader is directed to more specialized reports. We will then provide some (personal) perspectives on the evolution and potential future impacts of this field in chemical, cell, and molecular biology.

## 2 Antibody Structure Prediction

Antibodies are not only important investigative tools in (bio)chemistry, molecular biology, and medicine but are now largely employed as therapeutics in cancer, autoimmune and infectious diseases, and as diagnostic tools.^26−28^

Established antibody production processes rely on long and costly experimental procedures, often employing inoculated animals. This approach is sometimes limited by the capacity of animals to elicit antibodies targeting the desired epitope. In this framework, computational methods hold the potential of making antibody development more customizable, faster, and cheaper.^29^ Breakthroughs in computational structure prediction methods, high-throughput sequencing, and data analysis have indeed greatly contributed to *in silico* antibody development.^30,31^

Computational methods for antibody development entail structure prediction, design, and antigen–antibody complex characterization with the end goal of designing an optimal Ab sequence complementary to a desired antigen.

**Figure 1:**
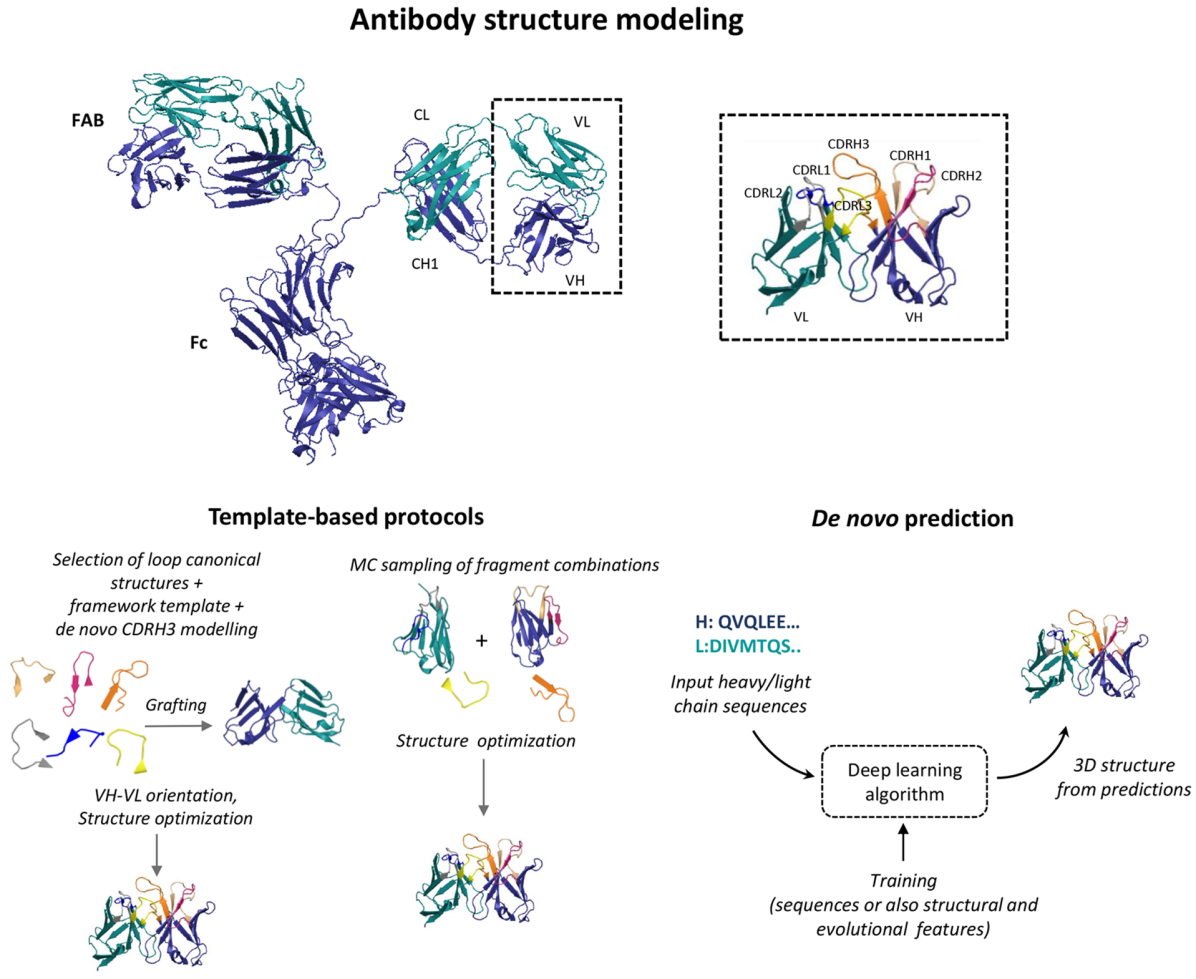
Antibody modeling and design. (Top) Full-sequence structure of an antibody and detail of VH/VL domains with CDR loop regions (PDB codes 1IGT(58) and 6OZB(59)). (Bottom) Overview of the antibody structure prediction methods. Template-based protocols rely on template construction from databases of fragments or framework regions onto which loop regions (usually obtained from canonical clusters) are grafted, followed by structural refinement steps. In some protocols the CDRH3 region is modeled *de novo*. In the figure, differences between two of the available protocols, Rosetta Antibody and AbPredict, are exemplified. *De novo* prediction methods can infer 3D structure from sequence and rely on deep learning algorithms trained on features encoding sequence and, in some cases, structural and evolutionary information.

Since immunoglobulins share a common Y-shaped fold, most traditional methods for modeling antibody structure rely on homology modeling strategies. Human antibody structure consists of two heavy and two light chains characterized by a well-conserved constant domain and the fragment antigen binding (FAB) domain, where conserved framework regions alternate with the highly variable loop regions named complementary determining regions (CDRs). The sequence/conformational variability in CDR regions, resulting from VDJ gene recombination and somatic hypermutation, is what makes affinity maturation and diverse antigen targeting possible. Yes, from the molecular designer’s point of view, this also represents one of the major challenges in antibody structure prediction. Fortunately, making the issue more manageable, canonical structural clusters of loop conformations/orientations have been established^32^ and usually, five of the six CDR regions fall into these representative structures. The same does not hold for the CDRH3 loop region, whose extended length and conformational variability make structural prediction more elusive. Small changes in heavy/light chain variable domain (VH-VL) orientations have also been shown to have a significant impact on CDR position and thus on antibody/antigen affinity. Therefore, efficacious modeling of the VH-VL interface is also a highly relevant matter. Figure 1 depicts an overview of the current strategies for antibody structural prediction, which will be described below.

Rosetta is one of the leading computational tools in biomolecular design and protein fold prediction. Initially developed in the group of David Baker at the University of Washington, it is now managed and developed by RosettaCommons, an international community counting more than 70 research groups.^33,34^ Among the many computational frameworks included in Rosetta, there are several protocols specific for the computational modeling and design of antibodies.^35,36^

Rosetta Antibody^37^ is a template-based protocol for structural prediction from sequence. First, selected templates for the framework and five canonical loops are grafted onto a preliminary model; at this point, the HCDR3 loop is modeled *de novo*, while the heavy/light chain variable region (VH-VL) orientations are refined. Template selection relies on a BLAST sequence search of the PyIgClassify database^38^ and includes the sampling of diverse VH-VL orientations. The CDRH3 *de novo* modeling step employs the next-generation kinematic loop closure algorithm (KlC) in increasing resolution steps together with side-chain packing and minimization. With time, more accurate conformational constraints were introduced to overcome the limited capacity of the modeling protocol to sample the so-called kinked conformations of CDRH3 which were, on the contrary, observed more frequently in antibodies’ native structures.^39,40^ Applicability to nonhuman or nonmurine antibodies is limited by the availability of structures in the PDB database. For such modeling tasks, templates could be entered manually or selected from a custom-made database. Similarly, the modeling of rare CDR conformations will be more reliable when more of those structures will be experimentally solved. Sphinx, a hybrid method combining *ab initio* and knowledge-based loop structural prediction, has been shown to outperform Rosetta.^41^

A second protocol for antibody modeling implemented in Rosetta is AbPredict,^42^ which does not rely on homologous templates. Experimentally determined antibody structures are fragmented into four backbone regions (light and heavy chain CDR3, heavy and light chain variable domains, which comprise the framework, and CDR1-2 region). These fragments are randomly recombined also considering different rigid-body VH-VL orientations to have a database of structures with a target sequence length. Starting from a randomly selected initial conformation, a Monte Carlo simulation is performed for sampling combinations of backbone fragments, packing of side chains, and minimization of the entire structure which outputs a single chain variable fragment (scFv) structure. Given the independence from sequence homology, it allows one to select templates with low sequence identity but high structural compatibility. On the other hand, the capacity of representing CDR loops of rare length is limited also due to the requirement that the target sequence and template match in length. The more recent implementation AbPredict2 is available as a Web server.^43^ Improvements consist of reduced computational cost, a decrease in the stereochemical strain of the model, and better sampling of light-heavy chain orientations.

Other open-source automated similar workflows are ABodyBuilder,^44^ Kotai Antibody Builder,^45^ and PIGS.^46^ ABodyBuilder is a fully automated computational pipeline comprising steps similar to those already described for Rosetta Antibody with template selection relying on sequence similarity, using FREAD^47^ for all CDR loops modeling and SCWRL4^48^ for side chains.^44^ It has the advantage of being a fast prediction tool, favoring high-throughput screenings, and implements a data-driven estimation of model accuracy as a measure of the probability of a region to be modeled within a defined threshold. Kotai Antibody Builder selects templates for framework and non-H3 CDR region, privileging statistically derived rules together along with sequence identity evaluation. For CDRH3 loop modeling, it relies on previously established rules for base region type prediction. Subsequent refinement can employ the SPANNER module for fragment-based structural prediction, followed by energy minimization steps and scoring.^45^ PIGS privileges template construction from a unique structure and relies on canonical structures for loop modeling.^46^ The main packages for full antibody modeling together with others that focus on more specific modeling tasks have been listed in a recent dedicated review.^30^

The AMA II benchmarking of many of these protocols revealed that their performance is overall similar, with an average RMSD of about 1.1 Å for the entire Fv and with the most critical region being the CDRH3 loop region, modeled with an average accuracy of about 2.2 Å but up to >5 Å RMSD in some cases.^49^

In addition to the above-described grafting-based tools, deep learning-based methods are emerging and are being heavily applied to fast and accurate antibody *de novo* structural prediction.^50,51^ Methods optimized for immunoglobulin structural prediction perform better than AlphaFold2, originally developed for general protein structural prediction.^52,53^ DeepAb, an improved version of DeepH3, was tested on the Rosetta Antibody benchmark and shown to outperform grafting-based methods.^54^ The method is based on a deep learning model trained using cross-entropy loss on pairs of heavy/light chain sequences derived from the Observed Antibody Space^55^ and can predict relative distances and orientations between pairs of residues of Fv domain from sequence information and previous information on Ab structural organization. The final step is a fast Rosetta-based protocol for the generation of the 3D protein structure. Insights about relevant amino-acid interactions and mutations improving binding affinity could also be derived. ABlooper,^53^ based on an equivariant graph neural network is currently the fastest method for predicting CDR conformations and was shown to achieve on average lower RMSD than ABodyBuilder but not DeepAb on models from the Rosetta Antibody Benchmark. The same group recently released IgFold which also embeds information from templates and has lower runtime for structural predictions.^56^ These methods circumvent the lack of experimentally solved structures on which grafting methods rely and, on the other hand, take profit from the large amount of sequence data currently available. Their short runtime (on the order of minutes for structural prediction) opens the possibility of high-throughput structural screenings. An extended description of AI-based methods is beyond the scope of this review. For further details, the reader is directed to other reports.^50,57^

## 3 Structure Prediction of Antibody–Antigen Complexes

**Figure 2:**
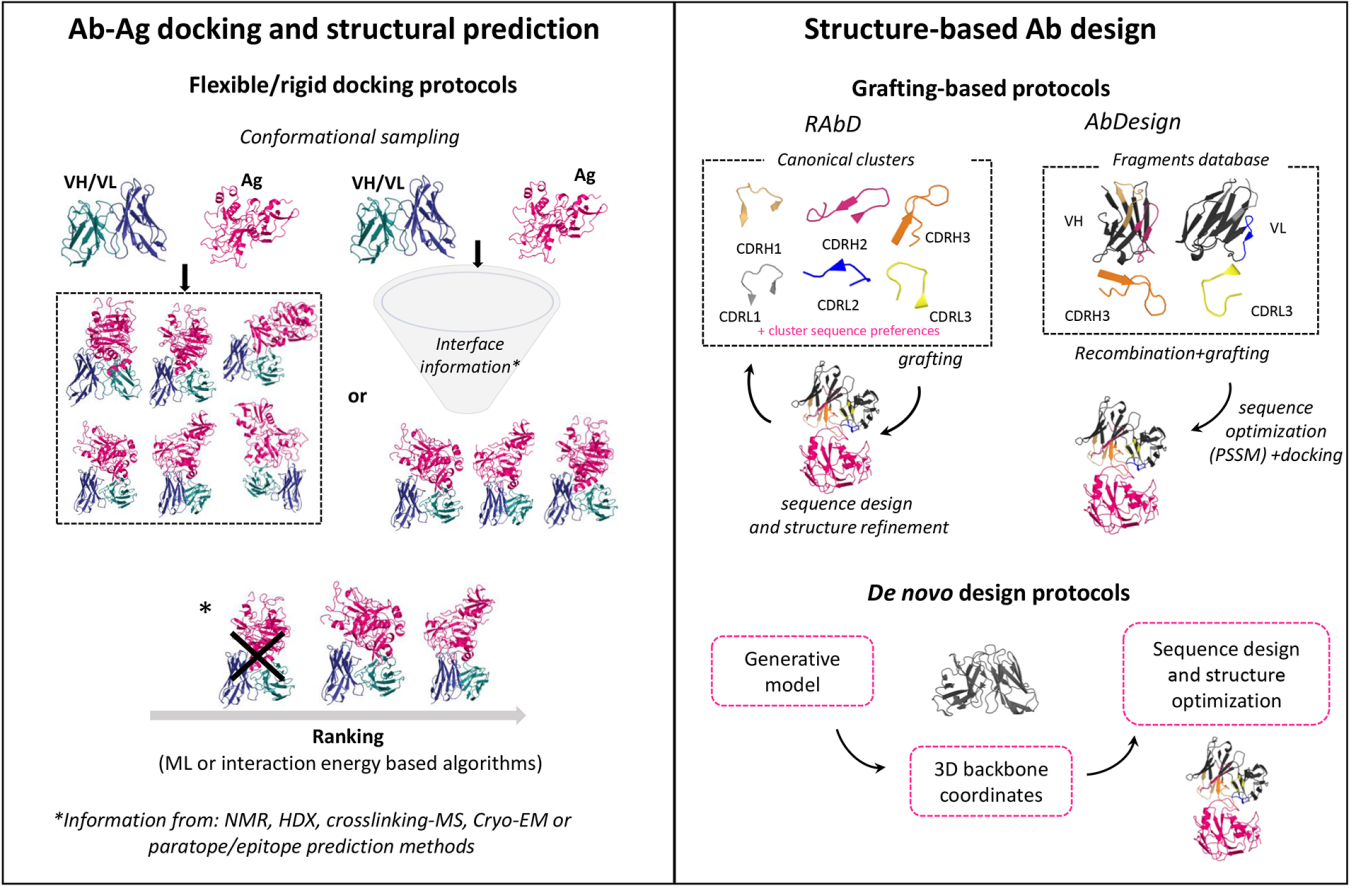
Antibody–antigen interactions and structure-based antibody design (left panel) overview of antibody antigen docking protocols. Rigid docking protocols only allow for rigid body movements of the binding partners whereas flexible docking protocols also include backbone or side chain refinement steps. Most of the docking programs envisage an integrative modeling approach meaning that information on the interface residues can be employed in the conformational sampling step or in the ranking/scoring step, or both. Structures are reproduced from PDB code 2Q8A.^26^ (Right panel) Summary of Antibody Design strategies. Rosetta Antibody Design and AbDesign are taken as illustrative examples of two different grafting-based protocols. Rosetta Antibody Design includes a routine for assembling of a template from a selected scaffold+CDR loop regions from a canonical cluster database followed by sequence and structure refinement in the presence of the binder. AbDesign builds a template from sampling of combinations of VH and VL domains (including all non-CDR3 loops) plus CDR3 regions followed by sequence optimization and docking onto the binder. *De novo* design protocols rely on generative models for obtaining 3D backbone coordinates, followed by sequence design and structural refinements. Structures are reproduced from PDB code 6OC3.^59^

While great improvements to apo-antibody structural prediction have been brought about by DL-based methods, reliable structural prediction of the antibody–antigen complex is still highly desirable for the future. Indeed, atomic-level structural information on antibody–antigen complexes is instrumental to investigate the interactions involved in forming the complex and providing a rational framework for the design of optimized interactors. Given the difficulty of directly predicting the multimer structures from sequences^60^ and of experimentally solving high-resolution structures, developing more and more accurate protein–protein docking methods is of paramount value. While general-purpose methods could be used, accurate prediction of the antibody–antigen interface is challenging given the conformational variability of the Ab loop regions involved. In an attempt to overcome such inherent challenges, tailored docking protocols were developed. They can be classified into rigid docking methods such as those implemented in ClusPro^61^ and ZDock,^62^ only allowing rigid body movements of the binding partners, and flexible/semiflexible methods that also include backbone/side chain refinement steps such as SnugDock,^37,63^ HADDOCK,^64^ SwarmDock,^65^ and LightDock.^66^ These tools implement an integrative modeling approach allowing the use of experimentally derived or sequence-conservation knowledge about the interface (Figure 2, left panel).^67^ Benchmarks of docking protocols were recently reported.^68,69^ The methods differ in their conformational space sampling strategies and in the way they employ previous knowledge: for instance, HADDOCK and LightDock employ information on the interface both for guiding the docking in the sampling step and for ranking and scoring the obtained models, whereas other tools like ZDock and ClusPro employ previous knowledge only in the scoring and filtering of the generated models. These aspects result in a high number of high-quality models when some knowledge is provided in the case of HADDOCK but a lack of good models when no good quality information is available. Other methods perform a more extensive sampling with a low percentage of near-native models. Thus, an accurate scoring function is needed in these cases. Improvements in the accuracy of the models come therefore from a more extensive knowledge of the interface or more accurate scoring functions/methods able to sort out near-native models from the generated poses. Experimental information about important residues or interface conformations can be derived, for example, from mutagenesis, cross-linking mass spectrometry, NMR or hydrogen–deuterium exchange experiments, Cryo-EM, and SAXS.^64,70−72^ In this context, knowledge derived from the computational prediction of paratopes and epitopes leveraging the great availability of sequence data is a very attractive resource in terms of its high-throughput potentialities and broad applicability. Epitope prediction remains the more challenging task and will be treated in detail in the next paragraphs (*vide infra*). Antibody-specific ML paratope predictors include PINet,^73^ PECAN,^74^ Parapred,^75^ proABC2,^76^ and others.

Another challenging aspect is that once docking poses have been generated, a good scoring function is needed for discriminating non-native from near-native complexes. In this regard, scoring functions can be derived more traditionally from interaction energy evaluation^77^ or also from statistical potentials obtained from known complexes. In the latter case, ML-based methods have been recently employed for improving pose ranking.^78,79^

## 4 Structure-Based Antibody Design

A computational problem complementary to the prediction challenges described above is antibody design: whereas structure prediction demands modeling of the protein fold starting from knowledge of the sequence, antibody design aims at defining the best sequence for a target 3D structure. The topic has been recently reviewed in depth elsewhere.^30,31^ In general, methods can be classified into *de novo* or grafting-based design protocols, and different criteria in the design process need to be adopted depending on whether the goal is to obtain a single antigen specific antibody or a protein capable of binding a broader spectrum of antigens (Figure 2, right panel).

Concerning the single-state design problem, Rosetta also includes antibody-tailored design protocols. RosettaAntibodyDesign (RAbD)^80^ is the design equivalent of RosettaAntibody. Starting from an antibody–antigen complex, first it is possible to design different CDRs from the canonical cluster database based on North–Dunbrack clustering^32^ and then the sequence is designed/optimized based on canonical CDR sequence preferences. Briefly, RAbD consists of a graft design routine which then passes the generated model to a protocol performing sequence design, side chain (re)packing, CDR minimization, and, if needed, docking with epitope- or paratope-based constraints. Minimization employs CDR cluster-derived constraints, and a Metropolis Monte Carlo criterion is used for optimization in both the graft design and subsequent steps. In this context, the total energy or interface energy can be considered as the guiding criterion. AbDesign^3,81^ applies to antibody design an approach similar to AbPredict: experimentally determined structures are fragmented analogously and then recombined and grafted onto the target structure. After constrained optimization of segments, dihedral and coordinate templates where each segment deviates <1 Å from the original structure are kept for subsequent sequence optimization according to conformation-dependent position-specific scoring matrices (PSSMs) for each segment. At this point, the predicted apo structure is docked against the target antigen to allow further backbone conformation/sequence optimization. Designs are scored based on different criteria concerning protein structure quality and shape complementarity/binding affinity with the antigen. Benchmarking of the method highlighted issues related to the expression of the designed antibodies and the deviation of the experimentally determined structure from the predicted one, indicating that rounds of optimization were required.

Protocols for multistate-design tasks aiming at the development of antibodies that can bind to multiple antigens, optimizing specificity through negative selection, or binding to different antigen conformations are also implemented. Broadly neutralizing antibodies have the potential of retaining neutralizing efficacy despite the escape mechanisms engaged by pathogens by targeting conserved residues.^82^ A suitable protocol is RECON MSD, which has the goal of optimizing the sequence so that it can adopt various conformations for binding more antigens or more conformational states of a single antigen.^83^ BROAD is an implementation that reduces computational costs.^84^

Vendruscolo and collaborators recently reported a novel strategy for designing antibodies that target epitopes whose structure is known (computationally or experimentally).^85^ Starting from the target epitope structure, the so-called AbAg database comprising linear CDR-like motifs and antigen-like sequences, derived from PDB structures, is searched for complementary CDR-like fragments. After CDR-like fragments are combined, contacts with the epitope are optimized, and CDRs are grafted onto an antibody scaffold. This approach circumvents the need for approximate energy calculations and sampling of the conformational and mutational space. The applicability is, of course, dependent on the availability of paratope-like fragments compatible with the target epitope.

A different approach to the structure-based antibody design computational problem was adopted by Huang and colleagues, resorting to the use of emerging AI methods.^86^ Ig-VAE is a Variational Autoencoder-based generative model that directly outputs 3D backbone coordinates that can then be combined with various constraint and optimization approaches for applications in antibody design. For example, this method was applied to the design of a SARS-Cov-2 ACE2 epitope binder. The latent space of this model can be exploited to expand the capacity of generating novel proteins that cannot result from the sampling of existing structures. The protein design problem is thus reformulated as a constrained optimization in the latent space of a generative model.

Another recent report moved in the direction of a generative model capable of codesigning the antibody CDR 3D structure and sequence, given a framework region restraint, through iterative refinement steps that optimize one in response to the other. This represents an advance since the optimal 3D antibody structure for a target antigen is seldom known *a priori*.^87^ A further important improvement to this model would come from applying the antibody design problem constraints and conditions derived from the stereoelectronic properties of the specific target antigen.

Similarly, other generative models such as RF Diffusion^88^ and Chroma,^89^ which have previously been applied to the design of protein binders hold the promise to bring advances to the Ab field.

The paradigm is thus shifting from an unconstrained structural generation complemented with a binding affinity predictor and conformational/sequence search to a constrained *de novo* structural generation of the antibody in the presence of the target epitope/antigen.

Currently, experimentally or computationally designed antibodies often need to undergo other rounds of optimization. In this regard, *in silico* methods that mimic natural affinity maturation have been developed. Both physics-based and ML-based methods aim at predicting the mutations that will enhance antibody stability and affinity for the antigen.^90−92^ Automated tools aiming at increasing antibody developability (i.e immunogenicity, solubility) can also be employed in the design process.^51^

In addition to classical antibodies, single-domain antibodies are also being developed since they present some advantages with respect to immunoglobulins. For instance, they may be easier to produce and manage and, given their reduced dimensions, they may encounter fewer adverse reactions when administered as biological drugs.^93−95^

## 5 Antigen and Epitope Discovery

### 5.1 Immunogen Discovery and Reverse Vaccinology

While antibody therapeutics are responsible for passive immunization, a vaccine is defined as a preparation capable of stimulating an active immune response in the host. Active immunity includes both the humoral response (antibody-mediated) and the cellular-mediated immune response.^96^ The ideal vaccine should cause a prolonged neutralizing immune response in the host and is composed of an antigen complemented with adjuvants.

Traditional vaccine discovery approaches relied on the direct investigation of pathogen components hypothesized to have a role in eliciting responses to identify protective antigens. This approach has led to the development of many lifesaving vaccines but has shown limitations in targeting infectious diseases or other pathologies characterized by immune evasion mechanisms. Examples are HIV, influenza, or malaria pathogens whose surface proteins elicit extreme sequence variation or are shielded by glycans (HIV, hepatitis C). The search for anticancer vaccines, a hot field of study at the moment, may also need different approaches.^97,98^

In 2000^99^ Rappuoli and colleagues spurred a dramatic change in the field by introducing the paradigm of Reverse Vaccinology, which has led to the development of a vaccine against meningococcus B. Taking advantage of whole genome sequencing, possible antigenic species are sorted out with the aid of immuno-bioinformatics tools, subject to different criteria such as surface exposition, conservation, toxicity, and allergenicity. The most promising species are then screened experimentally for their immunogenic activity. More recently, the term Reverse Vaccinology 2.0 has been coined to refer to the advancements brought to the field by the analysis of human protective antibodies, sequencing and cloning of B-cell repertoires together with structural characterization of antigens and epitopes.^97,100^ The investigation of effective antibody response allows the identification of promising corresponding antigens/epitopes.

Various open-source tools for complete RV workflows have been designed mainly for bacterial antigen discovery. They can be classified in filtering based methods such as Vaxign,^101^ NERVE,^102^ Jenner-predict,^103^ and VacSol,^104^ or machine learning-based methods including VaxiJen,^105^ Vacceed,^106^ Vaxign-ML,^107^ and the Bowman-Heinson^108,109^ method. Filtering-based methods give as a result putative vaccine candidates selecting them only based on predetermined threshold values on features like the probability of exposure to the immune system, to be an adhesion molecule or more generally involved in virulence, limited homology to host proteins and number of transmembrane domains predicted using external computational tools or databases (pSort, CELLO2GO, SPAAN, HMMTOP, BLASTp, OrthoMCL, DEG, Vaxitope, Pfam, VFDB).^110^

Machine learning-based methods are trained on a set of bacterial protective antigens and nonprotective proteins and are employed to perform a ranking of the probability of all searched proteomes being a protective antigen based on the predicted properties. VaxiJen classifies proteins based on physicochemical properties only and could be suitable for predicting also viral or cancer antigens. The Bowman-Heinson method employs a support vector machine classifier and was trained on a data set of bacterial protective antigens (BPAs) and non-bacterial protective antigens annotated with 10 features related to antigen properties.

A comparison of six of these RV tools (Vaxign, NERVE, Vac-sol, Jenner-predict, VaxiJen, and Bowman-Heinson) in the recognition of BPAs in 11 pathogens has been reported recently.^111^ Results highlighted that none of the methods recognized all the known BPAs, with the ML-based Heinson-Bowman method being the best performing according to the fraction of proteome identified as a good candidate and agreement with the testing set. Moreover, there is poor accordance between the outputs of the different tools, suggesting the need to interrogate more than one tool in antigen discovery research. Overall, the urge for improvements in protein annotation tools and incorporation in databases of proteins with negative experimental results to be employed as negative test sets for ML-based methods is highlighted.

Since this benchmark was reported, updated versions of the tested RV tools such as VaxyJen3^112^ and Vaxign2^113^ were implemented to address possible limitations. For instance, the Vaxign2 Web server includes a predictive framework and postprediction computational tools and can be also employed for the development of candidate viral vaccines. In the predictive part it incorporates both the filtering-based Vaxign and the ML-based Vaxign-ML^107^ tools. Postprediction analysis entails the prediction of MHC-I and II epitopes with MEME, epitope mapping onto the input proteins based on IEDB^114^ immune epitope database including B- and T-cell experimentally tested epitopes, calculation of population coverage, and prediction of protein function and orthologs.

### 5.2 Prediction of B-Cell Epitopes

Computational epitope prediction is of key importance in vaccine development. Indeed, humoral antibody-mediated immune responses rely on interactions between B-cell receptors and antigen epitopes, whereas cellular responses include, among others, T-cell mediated responses that rely on interaction between T-cell receptors (TCRs) and MHC-I and MHC-II epitopes present on antigen presenting cells. Different tools were developed for T- or B-cell epitope predictions.

B-cell epitope predictions can be classified as sequence-based or structure-based methods. The latter have been demonstrated to be more reliable,^115^ but their application is limited by the availability of 3D antibody–antigen structures. Indeed, B-cell epitopes are more frequently discontinuous (also named conformational epitopes), and their prediction poses great challenges to current predictive approaches, especially sequence-based ones that are more suitable for linear epitopes. Moreover, doubts have emerged on whether the question of searching for all the possible epitopes of an antigen for all possible antibodies is well posed.^116^ Some evidence suggests that in principle all exposed protein surface patches could represent an epitope, given the “right” interacting partner.^117,118^ Therefore, antibody-specific prediction methods were proposed.^119,120^ Clearly, these implicate that the target antibody is known, which is not always the case.

Recently, two sequence-based (Bepi-pred 2.0,^121^ CBTope^122^), some structure-based methods (SEPPA3,^123^ Disco Tope 2.0,^124^ ElliPro,^125^ EPSVR,^126^ BEpro,^127^ epitope3D^128^), and an antibody-specific B-epitope prediction method (EpiPred^120^) have been benchmarked.^116^ Results evidenced that while performances support the reliability and usability of the methods, they may be suboptimal most likely because they were trained on limited (and now potentially outdated) data sets.

To overcome current limitations, the authors suggest considering aspects that range from taking into account oligomerization properties, conformational changes, the possibility of incorporating residues that should be excluded from prediction, the prediction of glycosylated surfaces, the potential for other post-translational modifications, and introducing antibody sequences to guide epitope search.

In this framework, it has been shown that the introduction of protein language models for the representation of protein sequences can improve the performance of sequence-based methods in the prediction of both linear and conformational epitopes.^129,130^ The importance of training the models on updated larger data sets characterized by low redundancy was also pointed out. A further advance would be the incorporation of the target antibody in the prediction using the same encoding strategy of the antigen.

Overall, the role of structure-based computational approaches in the prediction of B-cell epitopes has significantly increased. Also, to improve the spectrum of properties of a newly discovered antigen, the fine physicochemical determinants of its immune reactivity need to be characterized. This is important for both applicative and fundamental research. From a fundamental point of view, this knowledge can unveil the basic mechanisms underlying molecular recognition mechanisms in immune responses. On the other hand, this would improve our capacity to design antigens or antigen mimics with optimized reactivities and pharmacological properties. Nowadays, structure-based methods can take advantage of advances in the accuracy of predicted protein structures and in the increasing number of experimentally solved structures of antibody–antigen complexes.

In this context, we have developed a structure-based method (BEPPE) for the prediction of interface regions in proteins based only on energetic determinants and available as a Web server.^131−134^ The method was proven successful for epitope prediction and reverse vaccinology applications.^135−138^

**Figure 3:**
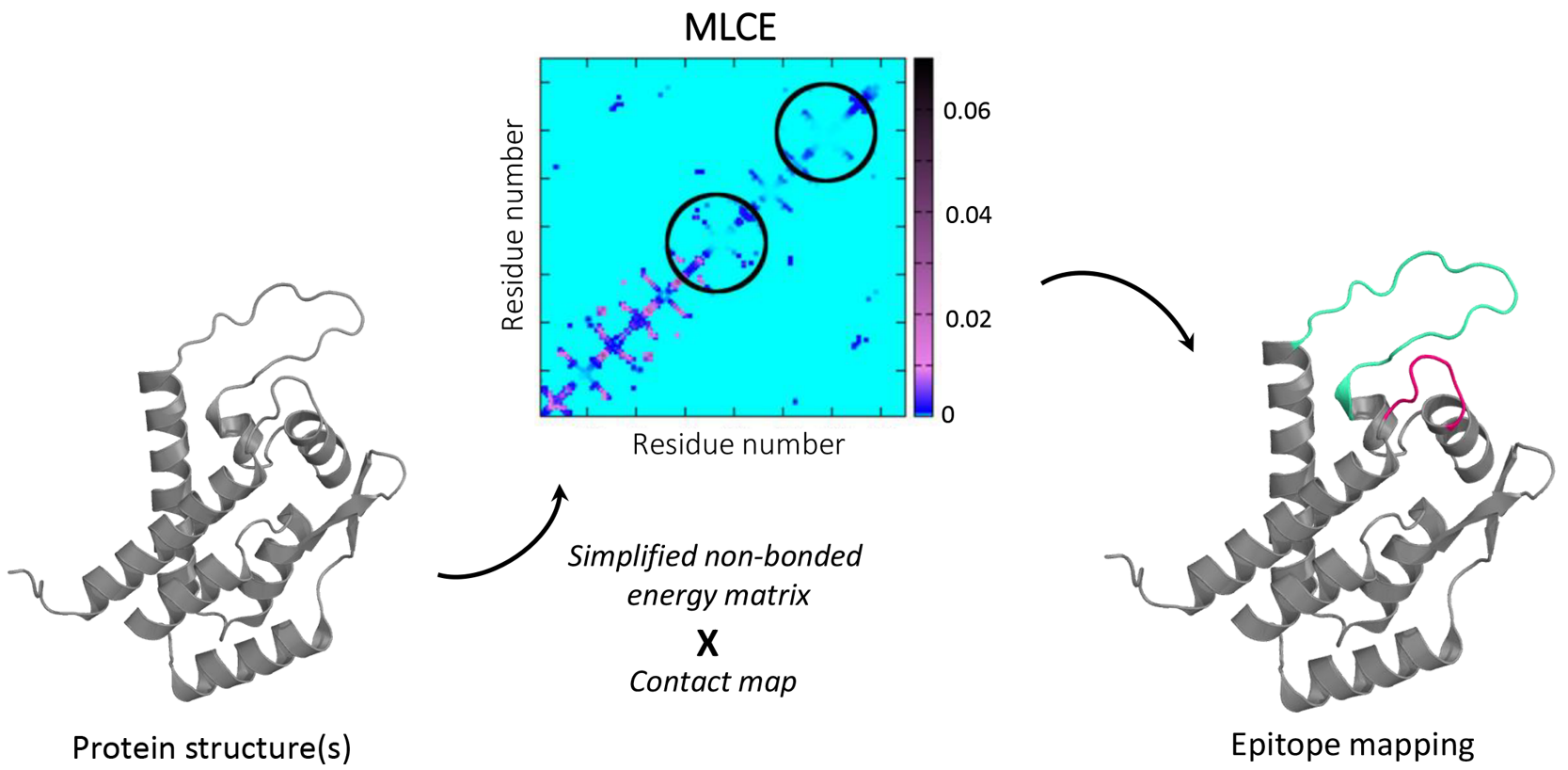
Epitope prediction. Schematic representation of the energy-decomposition-based epitope prediction method. The protein shown is reproduced from PDB code 1GWP.^26^

This prediction method is based on an energy decomposition approach: given a protein 3D structure (or also several structures extracted from MD simulations), the matrix (or average matrix) of pairwise nonbonded interactions is calculated. After eigenvalue decomposition, the most stabilizing eigenvector (or few eigenvectors)^139^ is employed to reconstruct a simplified matrix which is then filtered for retaining only local interactions and to obtain the so-called MLCE (matrix of local coupling energies). From this, patches that have the lowest energetic couplings with the surrounding structures are identified as putative interface regions with the potential to be recognized by Abs (see Figure 3). This method is based on the idea that epitopes (and interface regions in general) should be more prone to undergo conformational changes to interact with binding partners while contributing less to the overall protein stability and being mutation tolerant to facilitate escape from the host immune system.^140^

Another method relying on energy considerations was reported by Fiorucci and Zacharias.^141^ While other methods estimated desolvation penalties of protein surfaces by means of surface-area calculations, here the desolvation free energy penalty is obtained by probing the protein surface with a neutral low-dielectric sphere, therefore also considering the neighboring environment and long-range electrostatic effects. It was found that interface regions largely coincide with those characterized by lower free energy losses.^141^

Among ML-based partner specific methods, PINet, already mentioned as a paratope prediction tool, can predict antibody specific interface residues (both the paratope and epitope side) through a geometric deep learning approach aimed at dissecting the properties of complementary surfaces. It has been reported to have state-of-the-art performances in both antibody–antigen and protein–protein interaction cases.^73^

Recently, AxIEM has considered the issue of predicting “cryptic epitopes” of class I viral fusion proteins exposed in metastable conformations important for virulence and that are often the target of broadly neutralizing antibodies.^142^ The method leverages residue specific and environment features to determine the probability of a certain residue belonging to an epitope based on the conformation of the protein.

Interestingly, the SARS-CoV-2–human interactome was predicted with the computational pipeline PEPPI.^143^ It ranks interaction probability based on a naive Bayesian consensus classifier that combines outputs from independent modules that evaluate sequence and structural similarity to known protein–protein interactions, functional association, and a neural network classification. While results should be considered also based on other considerations not taken into account, such as subcellular colocalization of the predicted interactors, this and other similar tools can be useful for predicting relevant immunogens on a proteome-wide scale.

### 5.3 Prediction of T-Cell Epitopes and Neoantigens

T-cell epitope immunogenicity encompasses three fundamental steps: antigen processing resulting in the immunogenic peptide, binding of the peptide to MHC class I or II molecules on Antigen Presenting Cells (APCs), and binding of peptide-presenting MHC molecules (p-MHC) to TCR.

MHC class I molecules are expressed on the surface of all somatic cells and present peptides from the intracellular environment, whereas MHC class II molecules are found on immune cells such as B-cells, dendritic cells (DCs), and sample peptides from the extracellular milieu. CD8 T cells only interact with class I p-MHC and once primed and activated, they generate cytotoxic T cells. On the other hand, class II p-MHC complexes are recognized by CD4 T-cells that can give rise to different types of T helper cells. Among T-cell epitopes, neoepitopes are defined as those distinctive of cancer cells resulting from tumor-related genetic aberrations. The identification and targeting of neoepitopes is one of the most actively evolving fields of cancer immunotherapy aiming at the development of neoantigen based vaccines and T-cell based therapies.^144−148^ Current strategies for the identification of neoantigens rely on advanced sequencing techniques, including next-generation sequencing or -omics data aimed at individuating peptides distinguished from self-peptides. These are coupled with computational tools including those that we describe below for general purpose prediction of T-cell epitopes immunogenicity.^98,149−151^

Importantly, along with the general IEDB epitope database, T-cell epitope databases such as SYFPEITHY^152^ and ATLAS^153^ as well as the CEDAR cancer epitope database have been curated.^154^

**Figure 4:**
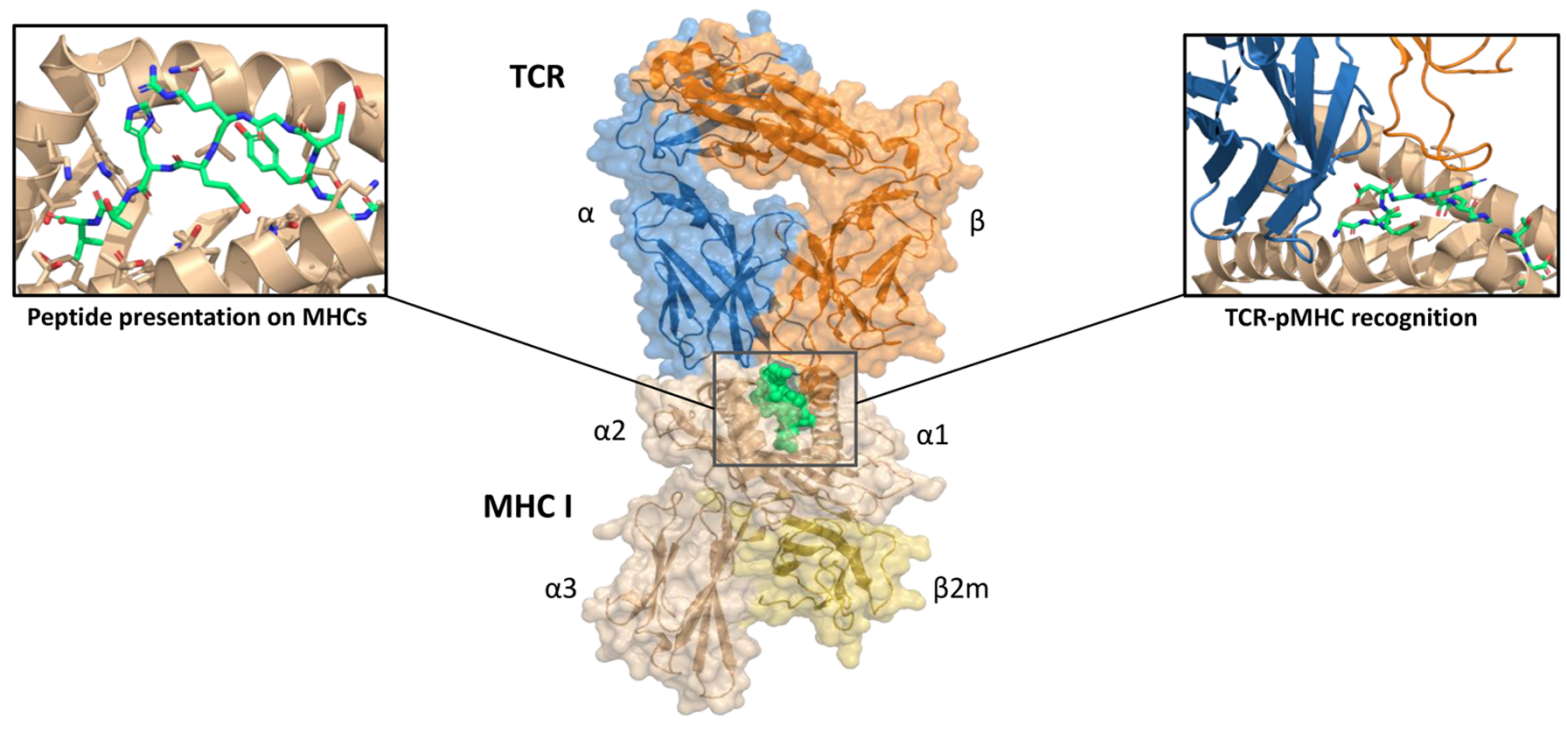
T-cell epitopes. Interaction between a peptide-presenting class I MHC molecule and a T-cell receptor, reproduced from PDB code 6TRO.^183^ After antigen processing, peptide immunogenicity is determined by its effective presentation on MHC molecules and binding of the pMHC complex by TCRs, which triggers downstream activation of CD4 or CD8 T-cells. Sequence- and structure-based computational tools for peptide binding affinity prediction and TCR-pMHC recognition are described in the text.

In this section, we will give an overview of computational tools predicting peptide binding to MHC molecules and effective interaction between p-MHC and TCR. Figure 4 depicts the interaction between a peptide presenting the MHC molecule and a T-cell receptor.

Binding to MHC I and II is similar but has some relevant distinctive features: MHC I groove is more closed, it typically hosts peptides ranging from 8 to 14 residues (more often 9), and binding site pockets have selective physical-chemical features. On the other hand, predicting binding to MHC II molecules is more challenging, as peptides are typically longer, even though only a 9-residue core typically sits in the more open binding groove featured with less discriminating interaction properties. A great number of sequence-based predictors have been developed that can either distinguish a binder from a nonbinder or also give an estimation of the binding affinity. These are listed in recent reviews^155,156^ and mostly rely on existing experimental elution or binding affinity data. While the first developed methods only took into account the presence of the so-called anchoring residues with suitable spacing, current methods consider fixed-length peptides (mainly 9 residues long) and quantify the contribution of each peptide position to the binding considering each residue independently in the case of linear methods (such as BIMAS),^157^ or also take into account nonlinear effects (for instance methods based on artificial neural networks (ANNs)^158^ or support vector machines (SVMs).^159^ Interestingly, it was demonstrated that algorithms taking into account nonlinear effects do not outperform linear ones in the prediction of binding to MHC class I molecules.^160^ The hypothesized explanation is that since peptides bind in the groove in an extended conformation, the contribution of each residue is dependent on its own interactions in the binding pocket with other nonlinear effects related to residue–residue interactions having a lower magnitude. Also, no significant gain of performance was obtained with prediction methods taking into account the 3D structure of the complexes with respect to sequence-based methods.^161^ However, MHC molecules in humans (called human leukocyte antigens (HLAs)) are characterized by great allelic variability. Indeed, different HLA molecules from different individuals will have different peptide preferences, and HLA supertypes with similar binding preferences were individuated and clustered. Since experimental data on which the above-mentioned models were trained are available for only a few allelic variants, they have a limited capability of predicting binding for all allelic variants. To overcome this issue, pan-MHC methods able to predict the binding of a peptide to uncharacterized HLA molecules have been developed. For example, ML models capable of performing such predictions for MHC I molecules such as NetMHCpan^162^ and a structure-based threading approach^163^ were developed by training the algorithm on information related to the MHC peptide binding site.^164^ Similar predictors for MHC class II molecules have been developed and improved versions of the initial models have been implemented more recently.^155,156,165^ Computational tools for predicting promiscuous sequences with high population coverage are also available.^166,167^ Interestingly, a method for predicting allele-specific anchor residue positions has been proposed for complementing neoantigen prioritization pipelines.^168^

Predictive tools have mostly taken into peptide presentation on MHC molecules and indeed, binding affinity to MHCs correlated well with immunogenicity for viral epitopes.^163^ However, the fact that good presentation on HLAs is necessary but not sufficient for stimulating T-cell response is particularly true for responses to neoantigens: owing to their endogenous nature, corresponding T-cells could have been deleted or tolerized to avoid self-immune reactions. Therefore, either mutations with respect to self-peptides should increase MHC binding (which is not so common) or (more likely) there should be structural differences in T-cell recognition with respect to wild type p-MHCs. In general, in the structural prediction of TCR-pMHC binding challenges are posed by the degree of TCR diversity and conformational flexibility of the TCR-pMHC interface. Therefore, at first, efforts were devoted to predicting TCR-pMHC from the protein sequences. Among sequence-based methods of notice is NetTepi^169^ which integrates previously developed methods for the evaluation of peptide-MHC stability (NetMHCstab) and binding (NetMHCcons), and a model for TCR affinity prediction reported by Calis and colleagues.^170^ The latter scores the peptide sequence based on rules derived from experimental data evidencing, for example, the relevance of positions 4–6. Other computational tools relying only on sequence have been described in more detail in a recent review.^171^ Sequence data driven methods have outlined some amino acidic physicochemical features related to immunogenicity such as the enrichment of aromatic hydrophobic and aromatic residues in the binding interface. However, physics-based and structure-based models can provide a rational background to these findings: for instance, the energetic gain of burying a hydrophobic residue by complex formation strongly depends on the 3D structural features. Given these premises, considering structural aspects alongside sequence holds the promise of giving better prediction of peptide immunogenicity and TCRs binding specificities.^172−174^

Baker and co-workers developed a neural network-based method trained on structural and energetic features derived from HLA-A2/peptide complexes.^175^ It outlines biophysical determinants of neoantigen immunogenicity such as mutations increasing the exposition of hydrophobic residues and overall peptide energy. Moreover, by including well-presented but not immunogenic as well as non-MHC binding peptides in the training data set it is able to capture both binding to HLA and TCR, thus predicting cases where low affinity to MHC is compensated by strong TCR binding and *vice versa*.

Modeling protocols specialized for pMHC-TCR structural prediction similar to those described for antibodies have been developed.^176−178^ Structural modeling was employed for optimizing force field-based scoring for evaluating the involved interactions. This modeling and scoring approach was employed to predict corresponding pMHC/TCR pairs given α and β subunits of MHC and TCR molecules and a pool of possible peptide binders.^179,180^ Very recently, a customized version of Alpha Fold specifically trained for pMHC-TCR modeling was employed for the same task with prediction success correlating well with 3D model accuracy.^181^

These results demonstrate recent advances in identifying interacting p-MHC and TCR among possible candidates, but further developments are needed for a more general prediction of the target peptides of a given TCR or if and which TCRs could bind to a novel epitope.^182^

## 6 Antigen Design

**Figure 5:**
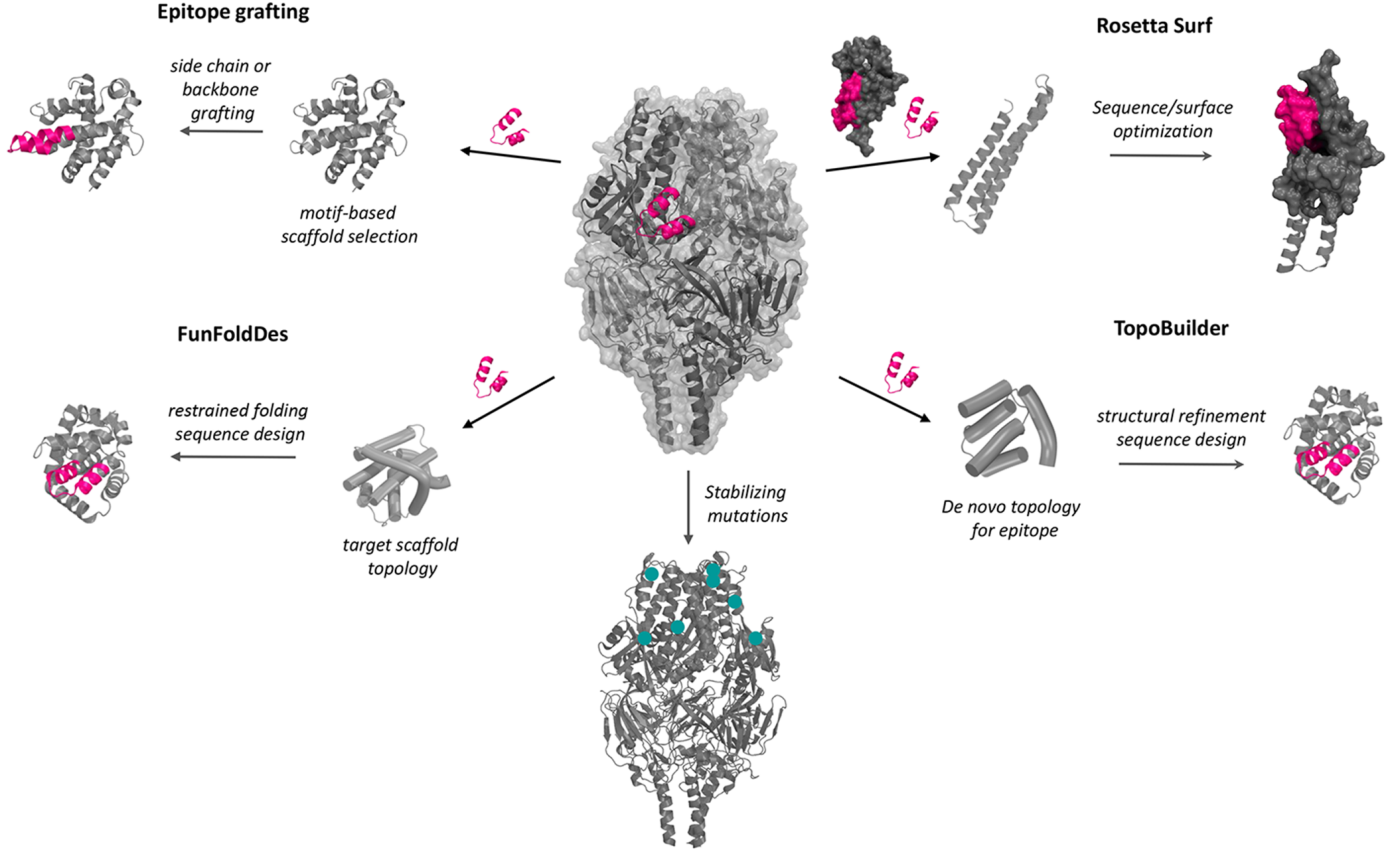
Overview of the main immunogen design approaches. Epitope grafting. Starting from a known epitope on the antigen, this can be transplanted through side-chain or backbone grafting onto a suitable scaffold chosen for structural compatibility with the motif to be transplanted. FunFolDes allows transplantation onto a defined topology instead of a specific scaffold protein; epitope grafting is followed by refolding of the rest of the protein into the target topology and sequence design. Rosetta Surf can follow the grafting of an epitope onto a scaffold and allows for surface optimization to mimic the native epitope environment and optimize complementarity with a target binder. TopoBuilder is a protocol for building *de novo* a topology around the epitope to be transplanted and has been complemented with FunFoldDes for the actual resulting protein design. Interestingly, these protocols allow biasing of the design process with the target binder. Another possible approach is to stabilize a particular conformation of the antigen exposing the desired immunogenic epitopes; an example are prefusion-stabilized variants of fusion proteins from viral pathogens.

With the advent of the new paradigms of reverse vaccinology 1.0 and 2.0, antigen/epitope design strategies have been envisaged to selectively present epitopes that are able to optimally elicit the desired immune response. The main design approaches are illustrated in Figure 5 and described in this section.

In this frame of thought, the idea of presenting only subdominant epitopes is related to the predominance of responses to immunodominant epitopes in the presence of the whole antigen species and was conceived to circumvent the capacity of pathogens (or malignancies) to mutate and/or mask immunodominant epitopes to evade the immune system.^184^ This approach may be particularly relevant in the development of vaccines for those diseases where traditional approaches are not effective such as HIV, respiratory syncytial virus (RSV), and cancer.

To this end, structure-based computational methods in the field of protein design have been implemented for the grafting of the desired epitope into novel scaffolds:^185^ the idea is to present the epitope in the context of a nonantigenic protein or of an already reactive antigen, thus generating a superantigen. This aim is more straightforward for linear epitopes, while it presents significant challenges for conformational discontinuous epitopes. Rosetta includes different protocols for the so-called epitope transplantation tasks.^36^ The first methods implemented allowed side-chain grafting when only side chains of binder interacting residues were grafted or backbone grafting when the entire peptide sequence was transplanted onto the scaffold. The choice of the scaffold protein was the critical step in that there should be a good superposition of the grafted motif backbone (side chain grafting) or C- and N-terminal regions (backbone grafting) and the scaffold to allow retaining the desired epitope conformation and overall protein stability. Our group developed the protocol SAGE, a computational pipeline that automates the prediction of the best grafting positions for linear epitopes onto a selected scaffold protein based on structure and sequence alignment, scoring of the secondary structure compatibility between the transplanted epitope and the scaffold, and scoring of surface exposition of the epitope.^186^

FunFoldDes follows previous approaches (i.e., FoldFromLoops) and involves the grafting of the epitope onto a scaffold topology instead of a specific protein, followed by structure folding (with some topology constraints) and sequence design.^187^ Importantly, the protocol also implements the possibility of biasing the design process in the presence of the binder. The latter outlines how proteins designed in the presence of the binder deviate from the accessible energy minimum of the protein fold and highlights the relevance of introducing “function constraints” in the process. This method is particularly useful for grafting loop regions for which proper protein scaffolds are more difficult to find and allows for a global adaptation of the protein backbone to motif incorporation. It has been employed for the design of scaffolds presenting antigenic site II of the respiratory syncytial virus fusion protein, which is a good proof-of-principle result. The resulting antigen was however shown to induce only low levels of site-specific neutralizing antibodies in nonhuman primates, indicating the space for additional optimization of the design approach.^188^ Applicability of this grafting strategy to more complex conformational epitopes remains difficult.

Within this frame, TopoBuilder is a protocol that reverses the computational pipeline: given the desired motif a stable protein is built around it as a carrier.^189^ First, a customized topology is assembled to accommodate the motif; then structural refinements and sequence design are performed with Rosetta FunFoldDes. This computational pipeline was applied for the design of a trivalent vaccine for RSV that combined the above-mentioned scaffold with the epitope from site II and two newly designed immunogens carrying structurally complex epitopes from sites 0 and IV.^189^ This construct was shown to induce an improved antibody response in immunized mice and nonhuman primates and importantly the possibility of guiding the induction of epitope-specific antibodies. Interestingly, the recently implemented protocol RosettaSurf^190^ allows sequence design for protein interfaces by employing information derived from the representation of the protein as a near-continuous surface featured by shape and electrostatic properties. After epitope grafting onto a novel scaffold, protein surfaces can then be compared by the implemented surface similarity or complementarity scores, which guide the surface/sequence design process. Importantly, the surface optimization step can be biased by the presence of a binder.

In general, such immunogen design tasks usually require the exploration of a great number of possible models and subsequent rounds of optimization guided by experimental results. Moreover, the scaffold presenting the desired epitope could also present some other new epitopes that could also cause some immunodominance issues that should be taken into account. Therefore, the current efforts now focus on improving or enhancing antibody responses. One possibility that is being explored is the use of nanoparticles as multivalent antigen carriers. Such nanosystems exploit inherent immune system features, optimized to detect exogenous particles. Indeed, for their shape and size they display advantageous biodistribution in the lymphatic system and uptake in lymphatic cells and can show increased immune response with respect to soluble antigens thanks to effects such as B-cell receptor cross-linking.^191,192^ Alongside more traditionally employed natural protein assemblies, self-assembling nanoparticles have been designed computationally by symmetrical docking of building blocks, followed by the design of protein–protein interacting surfaces.^193^ These nanoparticles were shown to be versatile and promising platforms for displaying several antigen molecules on the surface^193^ including heterotypic antigens^194,195^ leading the way to more customizable and easy-to-obtain platforms. The topic has been reviewed recently.^196−198^

Another immunogen design strategy includes the thermostabilization of known poorly stable antigenic proteins. Viral fusion proteins usually have a metastable prefusion conformation that rearranges after fusion of the viral envelope with the host cells and that often does not expose the epitope targeted by neutralizing antibodies. Therefore, stabilization of this class of proteins aims at retaining the immune-active prefusion conformations. These approaches have been employed for the development of prefusion stabilized forms of RSVF,^199^ SARS-Cov-2 spike protein,^200^ and HIV envelope.^201^ For achieving these results, structural knowledge has guided various rounds of optimization mostly relying on experimental techniques. Indeed, the application of computational structure-based methods for the design of thermostabilized immunogens seems still scarce, but as in the design of antibodies, there is great potential in the use of high-throughput protocols for the prediction of stabilizing mutations and in the computational screening of the properties of stabilized mutants. For example, PROSS is a tool available as a Web server that leverages information from proteins’ evolutionary sequence variability and is able to predict mutations that will increase protein stability based on ΔΔ*G* calculations.^202^

Molecular dynamics and free energy calculations have been employed to identify mutations increasing the stability of interactions between the pentameric subunits in the icosahedral capsid of the foot and mouth disease virus. This resulted in a safer experimental vaccine with an improved shelf life.^203^

The protein structures are reproduced from PDB codes 4JHW(204) (RSV fusion protein in its prefusion form), 2WH6,^205^ and 3LHP(206) (examples of scaffold proteins).

## 7 Conclusions and Perspectives

Structural immunology and vaccinology are among the research areas that are likely to benefit most from the current explosion in computing power and the advent of increasingly potent AI approaches.

The increase in computing power, combined with more and more efficient algorithms and improved force-field parameters, is already ushering in the era of whole-virion models and simulations. Approaches simulating multiple copies of an antigenic protein in a realistic environment have, in fact, recently started to appear. In the context of Covid research, the Hummer group, for instance, set out to identify possible antibody binding sites, with multimicrosecond molecular dynamics simulations of a 4.1 million atom system containing a patch of viral membrane with four full-length, fully glycosylated and palmitoylated S proteins. The authors combined the analysis of steric accessibility, structural rigidity, sequence conservation, and generic antibody binding signatures, to identify efficaciously the known epitopes on S. Moreover, this work revealed additional promising epitope candidates for structure-based vaccine design.^207^

The Amaro group recently reported impressive mesoscale, all-atom MD simulations of two evolutionary-linked glycosylated influenza A virions.^208^ The simulations reveal the main molecular motions of the principal surface antigenic targets of the influenza virus, hemagglutinin (HA), and neuraminidase (NA). Indeed, NA head tilting, HA ectodomain tilting, and HA head breathing are characterized at full atomistic resolution. The flexibility of NA reveals a cryptic epitope targeted by a novel convalescent human-donor-derived monoclonal antibody. Importantly, this type of work, combining extensive structural characterizations of the proteins in a realistic crowded environment and the possibility to analyze multiple copies of the same molecule in its native conditions, shows the possibility to obtain previously unappreciated (and inaccessible) views on the dynamics of antigenic proteins HA and NA. In this context, MD simulations could unveil transient intermediates able to induce antibodies against epitopes that, although conserved, are exposed only transiently in native molecules and may not be visible in a static 3D representation of the antigen. This, combined with the epitope prediction approaches described above, will help develop antibodies and vaccine components efficiently, for instance, by guiding the stabilization of specific conformations via mutagenesis.

Approaches like these will become more and more common as we begin entering the era of exascale computing. In this framework, efforts to adapt and port MD simulations to these new architectures will also play a key role.

Significant advances will likely stem from the integration of AI with computational biology and biotechnology for the development of new therapies and treatments.

One promising area of research is of course the use of AI to predict structures as well as design and optimize proteins as antigens, antigen-binders, and/or antibodies.^209,210^

If AI were to maintain its current promise, this may also find translation in the development of personalized medicine protocols. By analyzing a patient’s genetic data, medical history, and other factors, AI algorithms could predict which antigens will be most important to target and, as a consequence, which treatments are most likely to be effective for that individual.

In cancer treatment, AI supported interventions may be translated into the identification and molecular profiling of patient-specific cancer cells and then designing antibodies that can selectively bind to and destroy those cells while leaving healthy cells unharmed. Similarly, one could design novel and specific antigens that are able to elicit a strong protective immune response.

The combination of AI, protein design, and CAR-T cell therapy is an exciting area of research that has the potential to significantly improve cancer treatment outcomes. By identifying specific proteins or antigens that have specific signatures in cancer cells but not on healthy cells, researchers can design new CARs with improved safety and efficacy.

The same type of philosophy can be applied to the design of new proteins that can be used as CARs.

Clearly, limitations still have to be considered with a critical eye. Indeed, AI-based antigen and antibody design will depend on the correct understanding and modeling of the biophysical principles that underpin complex cell molecular recognition phenomena, a problem that is still far from being fully solved.

In this framework, it is important to note that similar to experimentally derived immunologically active molecules, unsolved issues remain. *In silico* developed antibodies, engineered T-cells, and immunogens could cause unwanted inflammatory reactions, toxicity, resistance phenomena, and other side effects. One advantage of (AI-powered) *in silico* screenings of increasingly rich databases is that they could help reduce or predict these risks. Given the complex interplay of the factors involved and the inherent variation of responses of different individuals, however, risks cannot be fully eliminated but can be mitigated by an improved knowledge of adverse response predictors. Furthermore, the computational design of immunologically active molecules remains difficult and usually involves several rounds of optimization with experimental testing to obtain molecules of good fitness and efficacy.

To advance along these fascinating avenues, an ever increasing integration of structural biology, computational biochemistry, computer and data science, and algorithm design will be necessary. New researchers and professionals that already embody this necessity are starting to appear. Importantly, novel (and at present mostly unpredictable) opportunities for the development of new research lines will begin to emerge.

We envision that the above-described developments will come of age in the next few years, opening new avenues for fundamental and applied research and facilitating the creation of new biomolecules with specific modes of action to be used both as therapeutics in the treatment of diverse diseases and as molecular tools to disentangle the intricacies of immune molecular mechanisms.
